# Clonal, Plasmidic and Genetic Diversity of Multi-Drug-Resistant Enterobacterales from Hospitalized Patients in Tripoli, Libya

**DOI:** 10.3390/antibiotics12091430

**Published:** 2023-09-10

**Authors:** Nada Elgriw, Véronique Métayer, Antoine Drapeau, Pauline François, Sana Azaiez, Maha Mastouri, Hajer Rhim, Adam Elzagheid, Najeeb Soufiyah, Jean-Yves Madec, Cherifa Chaouch, Wejdene Mansour, Marisa Haenni

**Affiliations:** 1 Department of Microbiology, Libyan Biotechnology Reseaerch Center, Tripoli P.O. Box 30313, Libya; nada.elgriw@btc.org.ly; 2 Faculty of pharmacy Monastir, Doctoral commission in Pharmaceutical Sciences, University of Monastir, Monastir 5000, Tunisia; maha.mastouri@rns.tn (M.M.); hajer.rhim@fpmh.u-monastir.tn (H.R.); cherifa.chaouch@fphm.rnu.tn (C.C.); 3 Laboratory of Transmissible Diseases and Biologically Active Substances LR99ES27, Faculty of Pharmacy, University of Monastir, Monastir 5000, Tunisia; 4 Unité Antibiorésistance et Virulence Bactériennes, ANSES—Université de Lyon, 69007 Lyon, France; veronique.metayer@anses.fr (V.M.); antoine.drapeau@anses.fr (A.D.); pauline.francois@anses.fr (P.F.); jean-yves.madec@anses.fr (J.-Y.M.); 5 Laboratoire de Recherche Biophysique Métabolique et Pharmacologie Appliquée, Faculté de Médecine Ibn Al Jazzar Sousse, Université de Sousse, LR12ES02, Sousse 4002, Tunisia; sana.azaiez@famso.u-sousse.tn (S.A.); wejdene.mansour@famso.u-sousse.tn (W.M.); 6 Department of Genetic Engineering, Libyan Biotechnology Reseaerch Center, Tripoli P.O. Box 30313, Libya; elzagheid@btc.org.ly; 7 Medical Microbiology and Immunology Department, Faculty of Pharmacy, University of Tripoli, Tripoli P.O. Box 13275, Libya; n.sufya@uot.edu.ly

**Keywords:** carbapenem, amikacin, colistin, *K. pneumoniae*, *E. coli*, nosocomial infection, NDM-1, OXA-48, KPC-2

## Abstract

Resistance to extended-spectrum cephalosporins (ESC) and carbapenems in Enterobacterales is a major issue in public health. Carbapenem resistance in particular is associated with increased morbidity and mortality. Moreover, such resistance is often co-harbored with resistance to non-beta-lactam antibiotics, and pathogens quickly become multi-drug-resistant (MDR). Only a few studies have been published on AMR in Libyan hospitals, but all reported worrisome results. Here, we studied 54 MDR isolates that were collected from 49 patients at the Tripoli University Hospital between 2019 and 2021. They were characterized using phenotypic methods, PCR and PFGE, and a sub-set of isolates were short- and long-read whole-genome sequenced. The results showed the frequent occurrence of *Klebsiella pneumoniae* (49/54), among which several high-risk clones were responsible for the spread of resistance, namely, ST11, ST17, ST101 and ST147. ESC and carbapenem resistance was due to a wide variety of enzymes (CTX-M, OXA-48, NDM, KPC), with their corresponding genes carried by different plasmids, including IncF-IncHI2 and IncF-IncR hybrids. This study highlights that implementation of infection prevention, control and surveillance measures are needed in Libya to fight against AMR.

## 1. Introduction

The international spread of multi-drug resistance in Enterobacterales has emerged as a major public health issue which, based on predictive statistical models, might have caused the death of 1.27 million people across the world in 2019 [1].

The proportions of resistance to extended-spectrum cephalosporins (ESC) have increased globally, mainly due to the spread of plasmid-borne extended-spectrum beta-lactamases (ESBLs) belonging to the CTX-M family. These enzymes have now penetrated all human, veterinary and environmental sectors, so that they are endemic in many parts of the world and considered a marker of antimicrobial resistance (AMR) burden in the One Health concept [2]. In Lebanon, for example, the ESBL carriage rate is already so high in the human community (between 34.5% and 52.9%) that there is a constant dynamic of colonization/decolonization of the healthy population by ESBL-producing Enterobacterales [3] that can potentially infect people, given the role of pathogens residing in patients’ microbiomes in the development of new infections [4].

Consequently, carbapenems stand as the first-line choice to treat severe infections due to ESBL-producing Enterobacterales. However, as bacteria adapt very quickly, carbapenemase-producing strains have also emerged over the last two decades, potentially leading to therapeutic failures in the worst cases [5]. It was also shown that carbapenem resistance led to longer hospital stays and higher mortality among inpatients with bloodstream infections in low- and middle-income countries (LMICs) [6]. The most frequent carbapenemases are now the OXA-48 and NDM enzymes, even though the VIM and KPC enzymes are also of concern. Moreover, carbapenemase-producing Enterobacterales are often multi-drug-resistant (MDR), potentially presenting resistance to other last-resort antibiotics such as colistin, fosfomycin or amikacin.

Libya is an upper–middle-income country with a population 6.7 million, whose healthcare system does not include systematic monitoring of AMR, either in the community or in hospitals. There are only a few publication on AMR in Libya, but they all report the frequent occurrence of carbapenem-resistant Enterobacterales that are possibly also resistant to colistin or amikacin. In 2011, six MDR *Klebsiella pneumoniae* (*K. pneumoniae*) were recovered from injured combatants who had been transferred to a Tunisian hospital [7]. They all presented the *bla*_OXA-48_ gene carried by an IncL plasmid, co-harbored the *bla*_CTX-M-15_ gene in five cases and the *bla*_CTX-M-14_ gene in the sixth one, and belonged to ST11, ST101 and ST147. Also in 2011, a similar *K. pneumoniae* ST101 displaying the *bla*_OXA-48_ and *bla*_CTX-M-15_ genes was identified in a Libyan refugee in Italy [8]. These two studies illustrate the fact that the *bla*_OXA-48_ gene is circulating in Libya and in its neighboring countries. In 2013–2014, 32 carbapenem-resistant *K. pneumoniae* were reported in two Tripoli hospitals but were not characterized molecularly [9]. A new level of resistance was reached in 2014–2015, with the emergence of *K. pneumoniae* belonging to different STs (ST11, ST15, ST101, ST147, ST405) and presenting the *bla*_OXA-48_ and/or the *bla*_NDM-1_ genes in a few cases, in addition to the *rmtC* gene conferring resistance to amikacin, or to insertions in the *mgrB* conferring resistance to colistin [10]. Finally, Slimene et al. reported, in 2020, 24 carbapenem-resistant Enterobacterales from a hospital in Sirte, including three *K. pneumoniae* OXA-48/NDM-positive ST383s and one KPC-positive ST640 [11], and in 2021, 76 carbapenem-resistant Enterobacterales from immunocompromised SARS-CoV-2 patients in Benghazi and Shahat (eastern part of Libya), which displayed various combinations of the *bla*_OXA-48_ and *bla*_NDM_ genes [12].

Several studies acknowledge the fact that data on the AMR burden are still limited in LMICs, even though this knowledge gap is recurrently highlighted [6,13,14]. There is an urgent need for data from LMICs in order firstly to improve surveillance in the countries themselves and allow the implementation of appropriate interventions, and secondly to provide the missing pieces needed to understand the emergence and spread of successful resistance genes, plasmids or clones. In line with this knowledge gap, our study followed three aims: first, to obtain up-to-date molecular information on ESC- and carbapenem-resistant Enterobacterales circulating between 2019 and 2021 in the Tripoli University Hospital (TUH); second, to describe the genetic determinants carrying these resistance genes, which have been largely overlooked in former publications from Libya; and third, to provide whole-genome data from Libya, to be compared with Libyan data that will be generated in the future or to already existing data from neighboring countries.

## 2. Results

Between 2019 and 2021, 44 non-duplicated *K. pneumoniae* and five *E. coli* were collected at the TUH, to which we added five *K. pneumoniae* isolates from the ancient collection dating from 2013. Since two to three different isolates were identified from four patients, 54 isolates collected from 49 different patients were further characterized (Table S1). Twenty-six patients were females (26/49, 53.1%) and seventeen were males (34.6%), while information was not available for six patients. They ranged in age from 1 day to 84 years, and 26 (53.1%) were children under 12 years of age. Most of the isolates originated from endotracheal tube tips (n = 18/54, 33.3%) and blood samples (n = 6, 11.1%). At least one resistant isolate was identified in each of the 18 wards studied, but the special care baby unit (SCBU; n = 18) and the pediatric intensive care unit (PICU; n = 7) were those in which most resistant isolates were retrieved (Table S1). Twelve different types of samples were collected, but resistant isolates were mostly found on the endotracheal tube (ETT) tips (n = 18) and in the blood samples (n = 6) (Table S1).

### 2.1. Genetic Diversity

**Figure 1:**
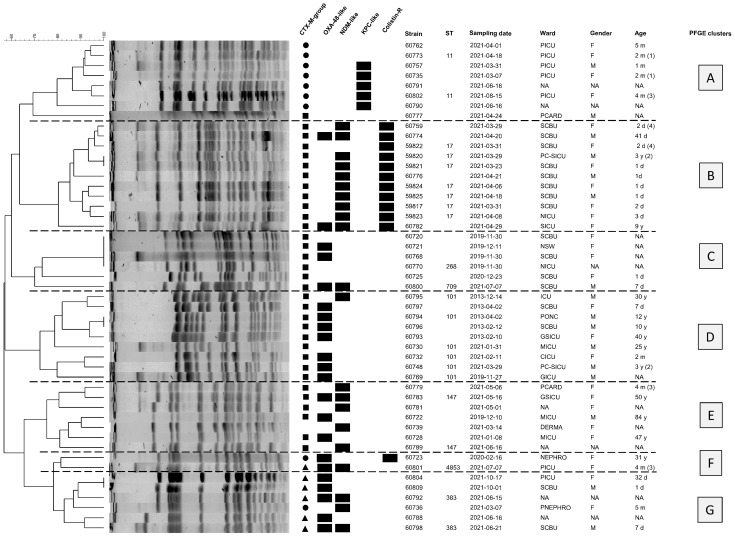
PFGE profiles and clustering of the *K. pneumoniae* isolates. Associated metadata are included, as well as the occurrence of resistance genes as detected via PCR (for CTX-M enzymes, the square indicates CTX-M-15, the circle CTX-M-65 and the triangle CTX-M15 + CTX-M-14). STs were included when available in the WGS data. The letters A to G define the different clusters of PFGE profiles.

The genetic diversity of all *K. pneumoniae* isolates was first assessed using PFGE (Figure 1). Seven main clusters of isolates (named A to G) could be delineated, among which six presented homogeneous profiles (A,B and D–G), while the last one (C) presented more diverging profiles.

Whole-genome sequencing was then performed on a subset of 21 of these *K. pneumoniae* isolates that were chosen to be representative of these seven different PFGE profiles. The six homogenous clusters could be assigned to ST11, ST17, ST101, ST147, ST4853 and ST383 while the last cluster presented two different STs, namely, ST268 and ST709. Of note, ST4853 is a *phoE* single-locus variant (SLV) of ST383.

For *E. coli*, five isolates presented different multiple-locus variable-number tandem-repeat (MLVA) profiles, while isolate #60731 presented the same profile and the same phylogroup as #60729. Consequently, only the four diverging isolates were whole-genome sequenced, which belonged to ST349 (two isolates), ST457 and the pandemic f*imH*30-ST131.

### 2.2. Resistance Phenotypes and Detection of Beta-Lactam Resistance Genes

All isolates displayed resistance to several families of beta-lactams, including to carbapenems in 65.3% of the *K. pneumoniae* isolates and 40.0% of the *E. coli* isolates, which were conferred by the presence of at least one beta-lactam resistance gene (Table 1).

A total of 115 genes belonging to 6 different families (*bla*_CTX-M_, *bla*_SHV_, *bla*_CMY_, *bla*_OXA-48_, *bla*_NDM_, *bla*_KPC_) were identified in *K. pneumoniae*, while eight genes (belonging to *bla*_CTX-M_, *bla*_CMY_ and *bla*_NDM_) were found in *E. coli* isolates (Table 2). Only three *E. coli* isolates and one *K. pneumoniae* isolate did not carry any *bla*_CTX-M-group_ genes. Ten isolates carried a *bla*_CTX-M-group_ gene alone, and the *bla*_NDM_ gene was found alone in one isolate (Table S1). Otherwise, all isolates displayed a combination of genes, including six isolates that harbored the three *bla*_CTX-M-group_/*bla*_OXA-48_/*bla*_NDM_ genes.

All isolates were also multi-drug-resistant, with resistance proportions being above 80% for kanamycin, sulfonamides, nalidixic acid and enrofloxacin in *K. pneumoniae*. Resistance to last-resort antibiotics such as amikacin and colistin was, respectively, identified in 12 isolates (10 *K. pneumoniae* and two *E. coli*) and 6 *K. pneumoniae* isolates, but no isolate carried resistance to both. Colistin resistance (>16 mg/L for all isolates) was due to an insertion in the *mgrB* gene, as proven by the modification of the size of the PCR amplicon, which moved from 230 bp to more than 1200 bp in all six resistant isolates. The hybrid assembly of isolate #60726 showed the presence of a 1219 bp fragment coding for an IS*3* family transposase (see below Section 2.4). The only antibiotics to which no resistance was observed were apramycin and florfenicol, which are not licensed for human use.

### 2.3. Genetic Characterization of the Resistance Genes

**Figure 2:**
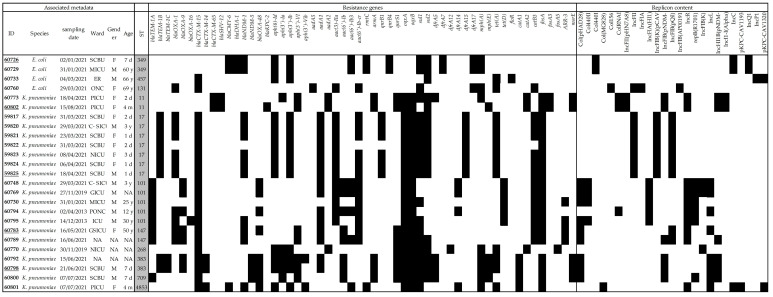
Heatmap of all resistance genes and replicons identified in all Illumina-sequenced isolates. Underlined IDs are those that were long-read sequenced. The presence of the gene is marked by a black square, and its absence by a white one.

Genomic analyses of the 21 *K. pneumoniae* and five *E. coli* fully sequenced isolates led to the identification of 56 different genes conferring resistance to a large number of antibiotic families and molecules (Figure 2). Different patterns of resistance could be seen depending on the different STs. All of CTX-M group 1 were subtyped as CTX-M-15; on the contrary, the CTX-M group 9 either belonged to the subtype CTX-M-65 when found with SHV-12 and associated with ST11, or to the subtype CTX-M-14 when found with CTX-M-15 in ST383 and its SLV ST4853. Only the *bla*_OXA-48_ variant was identified and the KPC gene belonged to *bla*_KPC-2_. On the contrary, two types of *bla*_NDM_ genes were found, namely, *bla*_NDM-1_ associated with ST17, ST147 and ST101 and *bla*_NDM-5_ associated with ST383, ST4853 and ST709. Finally, *bla*_CMY-6_ was found in two *E. coli* isolates.

Resistance to aminoglycosides was due to 14 different genes, which were preferentially found in one isolate or another depending on the bacterial species and ST. As an example, the *aadA1* gene was identified in the ST17 *K. pneumoniae* isolates, while *aadA2* was found in the ST11 isolates. Likewise, amikacin resistance was conferred by the *rmtC* gene in *E. coli* and by the *armA* gene in *K. pneumoniae*. At least one variant of the *qnr* genes (three were identified here) or the *oqxAB* genes was present in 23/25 of the sequenced isolates, conferring low-level resistance to quinolones. The common *sul1*/*sul2*, *dfr* and *tet*(A)/*tet*(D) genes were also frequently identified. The two ST11 isolates that were sequenced also carried the fosfomycin *fosA3* gene. Finally, no *mcr* genes were identified.

Fortunately, none of the *K. pneumoniae* strain presented important virulence factors, such as those coding for the regulator of the mucoid phenotype gene *rmp*A, yersiniabactin (*ybt* unknown), colibactin (*clb*3) or salmochelin operon (*iroBCDN*). Nevertheless, even though no isolate presented the intact aerobactin locus (*iucABCD-iutA*), five isolates presented the *iucC*-*iutA* genes, while four isolates presented the *iutA* gene alone.

### 2.4. Genetic Determinants Carrying the Beta-Lactam Resistance Genes

In order to decipher the genetic support of the beta-lactam resistance genes, five isolates representative of the diversity of the identified genes were long-read sequenced (Table 3).

*E. coli* #60726 carried the *bla*_NDM-1_ and *bla*_CMY-6_ genes on two different contigs. The *bla*_CMY-6_ gene was located on an IncC backbone, together with the *aac(6′)-Ib3*, *rmtC*, *aac(6′)-Ib-cr* and *sul1* genes. The *bla*_NDM-1_ gene was carried by a small element (>10,000 bp) that could not be assigned to any known replicon but has recurrently been published in an identical configuration, such as in pNDM-KN (accession number JN157804) recovered in 2009 from a clinical sample in Kenya [15]. *bla*_NDM-1_ is preceded by the IS*Kpn14* element and followed by the *ble*MBL, *trp1*-*dsbD* and *groES*-*EL* genes. This element was found inserted in IncA/C plasmids, which shared a very high homology with the contig2 found in our isolate (pNDM-US, accession number CP006661.1, collected in 2010 in the USA from a patient who had received medical care in India) [16]. This strongly suggests that #60726 carried a unique plasmid displaying the *bla*_NDM-1_ element inserted in the IncC plasmid.

*K. pneumoniae* #59825 carried the *bla*_NDM-1_ and *bla*_CTX-M-15_ genes. Two plasmids were identified, which belonged to the IncFIIk-IncFIB and IncFIBk replicons. The colistin resistance of this isolate was due to the interruption of the *mgrB* gene between nucleotides 46 and 47 by a 1219 bp DNA fragment coding for an IS*3* family transposase.

*K. pneumoniae* #60783 presented the *bla*_CTX-M-15_, *bla*_OXA-48_ and *bla*_NDM-1_ genes on two different plasmids. The *bla*_OXA-48_ gene was surrounded by two IS*4* family transposase IS*10A* genes and was carried by a typical IncL plasmid (63,600 bp) presenting no other resistance genes. The *bla*_CTX-M-15_ and *bla*_NDM-1_ genes were co-located on a small multi-drug resistance IncFIB plasmid (54050 bp) also harboring the *bla*_OXA-1_, *aac(6genes and was carried by a typical IncL plasmid)-Ib-cr*, *aph(3genes and was carried by a typical IncL plasmid)-VI*, *qnrS1*, *sul1* and *catB3* genes. This plasmid was highly similar (99% identity and 100% coverage) to the pSI0739-NDM plasmid (CP074090) identified in Italy, also in an ST147 *K. pneumoniae* [17].

*K. pneumoniae* #60798 also carried the *bla*_CTX-M-15_, *bla*_CTX-M-14_, *bla*_OXA-48_ and *bla*_NDM-5_ genes on two different plasmids. The *bla*_OXA-48_ gene was carried by a rather uncommon IncL plasmid (59,000 bp), which co-harbored the *bla*_CTX-M-14_, *aph(3″)-Ib* and *aph(6)-Id* resistance genes and resembled members of the pOXA-48-3 group described by Hendrickx et al. [18]. The *bla*_NDM-5_ and *bla*_CTX-M-15_ genes were co-carried on an additional large-size IncFIB/IncHI2 plasmid (360,100 bp) also displaying the *armA*, *aph(3′)-VI*, *aac(6′)-Ib*, *aadA1*, *qnrS1*, *sul1*, *sul2* and *dfrA5* genes. Such hybrid plasmids have already been reported to carry different resistance genes such as *mcr-1* or *bla*_NDM-5_ [19,20].

*K. pneumoniae* #60802 carried all three *bla*_CTX-M-65_, *bla*_SHV-12_ and *bla*_KPC-2_ genes on a unique IncF/IncR hybrid plasmid (126,000 bp), together with the *fosA3* gene. This plasmid highly resembled (99% identity and 98% coverage) the pIMI057-KPC plasmid (CP095423) found in an ST11 *K. pneumoniae* collected from a Chinese patient [21]. Of note, the formula of the IncF part of the hybrid plasmid was F33:A-:B-, and F33 plasmids have been shown to be major drivers of the dissemination of the *fosA3* gene, often associated with *bla*_CTX-M_ genes [22,23]. The #60802 isolate additionally displayed a larger IncFIB/IncHI2 hybrid plasmid (292,000 bp) carrying the *armA*, *aph(3′)-VI*, *sul2*, *qnrS1*, *msr(E)* and *mph(E)* genes, but no ESC or carbapenem resistance genes.

## 3. Discussion

*K. pneumoniae* is considered one of the major causes of global AMR burden, being among the top five most widespread nosocomial pathogens [24], and the World Health Organization (WHO) identified ESC- and carbapenem-resistant *K. pneumoniae* as a critical public health threat [25]. This study confirms that carbapenem-resistant *K. pneumoniae* are a major health issue in the Tripoli University Hospital, and most probably throughout the whole country according to the few publications on AMR in Libya [12]. Here, of the 54 isolates collected for their MDR phenotype, 49 belonged to the *K. pneumoniae* species, of which 35 presented at least one carbapenemase gene. Carbapenem-resistant *K. pneumoniae*, notably due to the reduction in treatment options, are also associated with high human morbidity and mortality [26]. This was the case here, since 10/44 patients (22.7%) included in our study died following infection with a carbapenem-resistant isolate, mostly (9/10) associated with contamination of the endotracheal tube tip.

The MDR or extensively drug-resistant (XDR) characteristic of *K. pneumoniae* is often restricted to a few successful high-risk clones that cause outbreaks internationally. The major *K. pneumoniae* high-risk clones encompass ST11, ST14, ST15, ST17, ST45, ST147, ST258, ST307 and ST512 [27]. In our collection, even though no ST258, ST307 or ST512 were found, high-risk clones were largely represented since several isolates belonged to ST11, ST17 and ST147. In particular, ST147 is well-known to be endemic in the Maghreb region, with several reports of carbapenem-resistant isolates in Algeria, Tunisia and Libya [24]. We also identified the ST101 clone, which is considered an emerging high-risk clone [28] and has been recurrently identified in the Mediterranean basin, just like ST147 [29]. The ST383 clone, which was particularly resistant here, concomitantly carrying the *bla*_CTX-M-15_, *bla*_CTX-M-14,_ *bla*_OXA-48_ and *bla*_NDM-5_ genes, was also identified in Egypt and China [30] and should be regarded as an emerging threat due to the convergence of resistance and virulence in certain isolates [31]. Interestingly, one of the five *E. coli* identified in this study belonged to the *fimH*30-ST131 clones, which are the most widely distributed *E. coli* clones, and commonly cause urinary tract infections.

Among the 49 patients included, 123 beta-lactam resistance-conferring enzymes were identified, mostly belonging to the CTX-M family (n = 68), followed by the OXA-48 (n = 21), NDM (n = 24), KPC (n = 5), CMY (n = 4) and SHV (n = 1) groups. Most of the isolates (44/54, 81.5%) presented at least two resistance genes, with predominance of the CTX-M/OXA-48 combination that was identified in 21 isolates. Finally, six isolates (11.1%) presented the combination CTX-M/OXA-48/NDM, showing that isolates tend to accumulate resistance genes even though they are largely redundant. All *K. pneumoniae* isolates presented alarming proportions of resistance to non-beta-lactam antibiotics, including to enrofloxacin (85.7%), sulfonamides–trimethoprim (83.7%) and gentamicin (65.3%), but also amikacin (20.4%) and colistin (12.2%). It is important to highlight that 5/10 patients who died carried a colistin-resistant isolate, evidencing the risk of treatment failure when such XDR isolates are infecting vulnerable patients. Amikacin resistance was due to the *rmtC* gene in *E. coli* and *armA* in *K. pneumoniae*, again illustrating the diversity of the genes circulating in the TUH. Fortunately, none of the isolates in our collection presented co-resistance of amikacin and colistin (but co-occurrence of the fosfomycin and amikacin resistance genes was observed) and none presented important intact virulence factors. However, the emergence of such worrisome isolates has to be closely monitored.

The beta-lactam resistance genes were all carried by diverse plasmids. The *bla*_OXA-48_ gene was identified on two different variants of the highly successful IncL plasmid, the most common one carrying only carbapenemase, and a second one additionally carrying the *bla*_CTX-M-14_, *aph(3*″*)-Ib*, and *aph(6)-Id* genes. Two MDR IncF-IncHI2 and IncF-IncR hybrid plasmids were also identified. It has been suggested that the fusion of two resistance plasmids might allow the non-conjugative plasmid to disseminate [32]. Another hypothesis is that unlike the chromosomal integration of CTX-M genes, which increases gene stability in non-selective conditions, the fusion of plasmids might increase the chances for the bacteria to become resistant in highly selective conditions. Nevertheless, in both situations, the transmission of AMR genes is facilitated.

The limitation of this study is that only resistant isolates were retrieved, so no information is available on the true prevalence of these pathogens in the intensive care units as well as in the other wards. This information is also not available from other publications on AMR burden in Libya. Future studies should thus fill in this knowledge gap in order to adapt measures in the fight against AMR. Likewise, concomitant studies on the proportion of ESC and carbapenem resistance outside hospitals are also needed to understand the routes of transmission of these pathogens in both hospitals and the community, and between these two settings. Also, this study focused on ESC- and carbapenem-resistant Enterobacterales, thus neglecting *Acinetobacter baumannii*, *Pseudomonas aeruginosa*, Gram-positive pathogens or anaerobes, among which are important pathogens contributing to the burden of AMR. In contrast, the strength of this study is that it provides the scientific community with molecular information on the genes, plasmids and clones that are circulating in Libya, as a basis for future studies and comparisons.

## 4. Materials and Methods

### 4.1. Study Design and Bacterial Isolation

A prospective study was conducted in Tripoli University Hospital (TUH), Libya, between 2019 and 2021. TUH is a tertiary care hospital containing 1400 beds. All Enterobacterales presenting resistance to ESC and carbapenems (as determined by the microbiology laboratory of the TUH using a VITEK **^®^**2 System v06.01 (bioMérieux)) and isolated in 18 wards of the TUH (including the general and pediatric intensive care units, as well as the emergency room and the dermatology, neuro-surgery, nephrology and oncology units) were kept at −80 °C for further characterization. Collected isolates were re-inoculated on MacConkey agar plates supplemented with imipenem or cefotaxime 2 µg /mL, and one colony per morphology was picked up from each selective plate. Identification was performed using VITEK **^®^**2 GN cards.

When available in the patients’ files, demographic data (age, gender, origin) were recorded, together with the origin of the sample, the isolation date, the treatment and the treatment outcome. The consent of the patients or that of their guardians was obtained for each collected sample.

For comparison purposes, only the five ESC-/carbapenem-resistant isolates found in the TUH collection and dating back to 2013 were included in the study.

### 4.2. Antimicrobial Susceptibility Testing

Susceptibility testing was performed on all 49 non-duplicate *K. pneumoniae* and five *E. coli* using the disc diffusion method on Mueller–Hinton agar, according to the guidelines and clinical breakpoints of the Antibiogram Committee of the French Society for Microbiology (CA-SFM; www.sfm-microbiologie.org). The *E. coli* ATCC 25922 strain was used as quality control. A total of 16 β-lactam (amoxicillin, piperacillin, ticarcillin, amoxicillin/clavulanic acid, piperacillin/tazobactam, ticarcillin/clavulanic acid, cefalotin, cefuroxime, cefotaxime, ceftiofur, ceftazidime, cefoxitin, cefepime, cefquinome, aztreonam and ertapenem) and 14 non-β-lactam (tetracycline, kanamycin, tobramycin, gentamicin, amikacin, apramycin, netilmicin, streptomycin, florfenicol, chloramphenicol, sulfonamides, trimethoprim, nalidixic acid and enrofloxacin) antibiotics were tested (Mast Diagnostics). Extended-spectrum beta-lactamase (ESBL)-producing Enterobacterales were detected using the Double Disc Synergy Test (DDST). Minimum inhibitory concentrations (MICs) were determined via broth microdilution for colistin, according to the European Committee for Antimicrobial Susceptibility Testing (EUCAST).

### 4.3. Detection of Resistance Genes and Strain Clonality

The *bla*_CTX-M_ genes were detected using CTX-M group-specific multiplex PCR [33]. The presence of the carbapenemase genes *bla*_OXA-48_-like, *bla*_NDM_ and *bla*_KPC_ was detected via multiplex PCR [34]. The presence and the size of the *mgrB* gene was investigated via PCR in all *K. pneumoniae* isolates [35].

The major *E. coli* phylogenetic groups (A, B1, B2, or D) were identified according to Doumith et al. [36]. The MLVA profiles of all *E. coli* were determined according to a published protocol [37].

PFGE was performed on all *K. pneumoniae* isolates using the restriction enzyme *Xba*I. Electrophoresis was conducted via a CHEF Mapper XP system using 6V/cm at 14 °C for 24 h, with pulse times ramping from 10 to 60 sec using an angle of 120°, as already described [38]. PFGE results were interpreted according to international recommendations [39].

### 4.4. Illumina Short-Read Sequencing and Data Analyses

DNA was extracted using the NucleoSpin Microbial DNA extraction kit (Macherey-Nagel, Hoerdt, France) and sequencing was performed using a NovaSeq 6000 instrument (Illumina, San Diego, CA, USA). Quality control of the reads was performed using FastQC and low-quality sequences were trimmed using Trimmomatic v0.39. De novo assembly was performed using Shovill v1.0.4 and the quality of assemblies was assessed using QUAST v5.0.2 (Table S2). Identification was performed using Kraken (https://github.com/DerrickWood/kraken). Sequence types (STs), resistance genes and virulence factors were determined using the CGE online tools (http://www.genomicepidemiology.org/) MLSTFinder v2.0, ResFinder v4.1 and VirulenceFinder 2.0.3, while replicon content and plasmid formula were identified using PlasmidFinder 2.0.1 and pMLST 2.0.

### 4.5. MinION Long-Read Sequencing

MinION long-read sequencing libraries were prepared according to Oxford Nanopore Technologies using the native barcoding expansion kit (EXP-NBD104) and the ligation sequencing kit (SQK-LSK109). Sequencing was performed on a MinION sequencer using a SpotON Mk 1 R9 version flow cell (FLO-MIN106D). Assembly of both Illumina and Nanopore reads was performed using Unicycler. The assembled contigs were annotated using Bakta (Web version 1.7.0/ DB: 5.0.0) [40].

### 4.6. Characterization of Plasmids

The genetic determinants carrying the ESBL/AmpC genes were detected via Southern blot on S1-digested DNA PFGE gels as already described [38], using the DIG DNA Labeling and Detection Kit (Roche Diagnostics, Meylan, France) according to the manufacturer’s instructions. In parallel, information on plasmids was extracted from the Illumina short-read and MinION long-read sequences as described above.

## 5. Conclusions

The occurrence of ESC- and carbapenem-resistant Enterobacterales, among which *K. pneumoniae* and, to a lesser extent, *E. coli*, have been evidenced by our study in Tripoli, and by Slimene et al. in Sirte, Benghazi and Shahat [11,12], strongly suggests that these pathogens have widely disseminated in hospitals throughout Libya. Our results showed that resistance to last-resort antibiotics (ESC, carbapenems, amikacin, colistin) is conferred by a large variety of genes, plasmids and clones, thus making their transmission more efficient. Nosocomial infections caused by MDR/XDR *K. pneumoniae* or *E. coli* are associated with high rates of morbidity and mortality, so that the implementation of infection prevention, control and surveillance measures is needed to avoid further dissemination of multi-drug-resistant clones.

## Figures and Tables

**Table 1:** Antimicrobial resistance profiles of the 49 *K. pneumoniae* and five *E. coli* from Libyan patients.

	*K. pneumoniae* (n = 49)		*Escherichia coli* (n = 5)	
	n	%	n	%
Amoxicillin ^1^	−	−	5	100.0
AMC ^2^	39	79.6	3	60.0
Cefalotin	48	98.0	5	100.0
Ceftiofur	47	95.9	5	100.0
Cefuroxime	48	98.0	5	100.0
Ceftazidime	45	91.8	5	100.0
Cefquinome	48	98.0	4	80.0
Cefepime	44	89.8	4	80.0
Cefoxitin	41	83.7	3	60.0
Ertapenem ^3^	32	65.3	2	40.0
Streptomycin	6	12.2	4	80.0
Kanamycin	43	87.8	4	80.0
Gentamicin	32	65.3	3	60.0
Tobramycin	39	79.6	4	80.0
Netilmicin	38	77.6	4	80.0
Apramycin	0	0.0	0	0.0
Amikacin	10	20.4	2	40.0
Tetracycline	33	67.3	2	40.0
Chloramphenicol	18	36.7	1	20.0
Florfenicol	0	0.0	1	20.0
Colistin ^4^	6	12.2	0	0.0
Sulfonamides	41	83.7	5	100.0
Trimethoprim	37	75.5	4	80.0
Nalidixic acid	42	85.7	2	40.0
Enrofloxacin	42	85.7	2	40.0

^1^ Not tested for *K. pneumoniae* as it is an intrinsic resistance. ^2^ AMC = amoxicillin + clavulanic acid. ^3^ Three *K. pneumoniae* isolates (#60730, #60762 and #60773) were resistant according to disc diffusion in the absence of carbapenemase genes. MIC determined via E-test proved that these isolates were susceptible to ertapenem and were thus not counted here. ^4^ As tested by MIC (broth dilution).

**Table 2:** Beta-lactam resistance genes identified in the 49 *K. pneumoniae* and five *E. coli* from Libyan patients.

	*bla*_CTX-M group 1_	*bla*_CTX-M group 9_	*bla*_CTX-M group 1 + 9_	*bla*_SHV-12_	*bla*_OXA-48-like_	*bla*_NDM-like_	*bla*_KPC-like_	*bla*_CMY-like_	Total
*K. pneumoniae*	36	24	6	1	21	21	5	1	115
*E. coli*	2	0	0	0	0	3	0	3	8

**Table 3:** Characteristics of the plasmids identified using long-read sequences.

Isolate	Species	ST	Long-Read Contig	Beta-Lactam Resistance Gene	Additional Resistance Genes	Replicon Type	Size (bp)
59825	*K. pneumoniae*	17	contig 2 contig 3	CTX-M-15 NDM-1, CTX-M-15	TEM-1, SCO-1, *aadA*, *aac(3)-IIa*, *aph(6)-Id*, *sul2*, *dfrA15*, *tet(A)* TEM-1, OXA-1, *aac(6′)-Ib-cr*, *aac(3)-Iia*, *qnrB1*,	IncFIBk IncFIIk, IncFIB (pQil)	253,391 128,170
60783	*K. pneumoniae*	147	contig 2	OXA-48	none	IncL	63,600
			contig 3	NDM-1, CTX-M-15	*aac(6′)-Ib-cr*, *aph(3′)-VI*, *qnrS1*, *sul1*, OXA-1, *catB3*	IncFIB (pQil)	54,050
60798	*K. pneumoniae*	383	contig 2	NDM-5, CTX-M-15	*armA*, *aph(3′)-VI*, *aac(6′)-Ib*, *aadA1*, *qnrS1*, *sul1*, *sul2*, *dfrA5*	IncFIB/IncHI2	361,000
			contig 3	OXA-48, CTX-M-14	*aph(6)-Id*, *aph(3″)-Ib*	IncL	59,000
60802	*K. pneumoniae*	11	contig 3	KPC-2, SHV-12, CTX-M-65	fosA3	IncF/IncR	126,000
60726	*E. coli*	349	contig 2 contig 3	CMY-6 NDM-1	*aac(6′)-Ib3*, *rmtC*, *aac(6′)-Ib-cr*, *sul1* none	IncC No hit found	127,679 9924

## Data Availability

This project was deposited in DDBJ/EMBL/GenBank under the BioProject accession number PRJNA998204.

## Supplementary Materials

The following supporting information can be downloaded at: https://www.mdpi.com/article/10.3390/antibiotics12091430/s1, Table S1: characteristics of the 54 isolates detailed in this manuscript; Table S2: quality control of all WGS data.
